# Transarterial Infusion Chemotherapy and Embolization for Patients With Unresectable Advanced Cancer of Stomach or Gastroesophageal Junction: A Retrospective Study

**DOI:** 10.1002/cam4.70396

**Published:** 2024-11-05

**Authors:** Lingqiang Min, Zheng Liu, Bo Zhou, Peng Zhou, Rongkui Luo, Yuqin Ding, Yuehong Cui, Zhongyi Shi, Yuan Gu, Yihong Sun, Zhaoqing Tang, Xuefei Wang

**Affiliations:** ^1^ Department of Emergency Surgery Zhongshan Hospital, Fudan University Shanghai China; ^2^ Shanghai Medical College Fudan University Shanghai China; ^3^ Department of Gastrointestinal Surgery Zhongshan Hospital, Fudan University Shanghai China; ^4^ Department of Interventional Radiology Zhongshan Hospital, Fudan University Shanghai China; ^5^ Department of Pathology Zhongshan Hospital, Fudan University Shanghai China; ^6^ Department of Radiology Zhongshan Hospital, Fudan University Shanghai China; ^7^ Department of Medical Oncology Zhongshan Hospital, Fudan University Shanghai China; ^8^ Department of General Surgery Zhongshan Hospital, Fudan University Shanghai China; ^9^ Gastric Cancer Center Zhongshan Hospital, Fudan University Shanghai China

**Keywords:** retrospective study, transarterial infusion chemotherapy and embolization, unresectable gastric cancer

## Abstract

**Purpose:**

The feasibility of transarterial infusion chemotherapy and embolization (TAICE) in the treatment of advanced gastric cancer remains unclear. This study explored the value of TAICE in patients with unresectable locally advanced or metastatic cancer of stomach or gastroesophageal junction (GEJ).

**Methods:**

Patients with unresectable gastric cancer who received TAICE for tumor hemorrhage cessation were enrolled in this retrospective study. TAICE was performed using the Seldinger method. The tumor feeding artery was selected for infusion chemotherapy and then was embolized by microspheres or gelatin sponge. Patients involved in this study received one to four cycles TAICE with one to three drugs in the regimen. The possibility of surgery was evaluated after TAICE. Objective response rate (ORR), disease control rate (DCR), R0 resection rate, pathological complete remission (pCR) rate, major pathological remission (MPR) rate, progression‐free survival (PFS), overall survival (OS), and safety were analyzed.

**Results:**

Between January 2015 and December 2020, a total of 27 patients received a median of 2 (range, 1–4) cycles of TAICE. ORR and DCR were 33.3% and 74.0%, respectively. Eighteen patients received surgery, and 15 of them underwent gastrectomy and D2 lymph node dissection, with an R0 resection rate of 83.3% (15/18). Four (26.7%, 4/15) patients achieved MPR, but none achieved pCR. The median PFS was 19.8 months (95%CI, 12.1–40.0), and the median OS was 36.1 months (95%CI, 21.0–not reached). Patients with gastrectomy had significantly longer PFS (40.0 vs. 9.5 months, *p* < 0.0001) and OS (not reached vs. 16.6 months, *p* < 0.0001) than those without gastrectomy. All the TAICE‐related adverse events were manageable, with the most common being fatigue (100%), nausea (63.0%), and vomiting (55.6%). No severe surgical complications occurred.

**Conclusion:**

TAICE was well‐tolerated and could be a potential therapy to provide opportunity of surgery for patients with unresectable advanced gastric or GEJ cancer.

## 1 Introduction

Gastric cancer is the fifth most common malignancy and the third‐leading cause of cancer‐related mortality worldwide [1]. Gastric cancer contributes to a high burden of disease in China for more than half of gastric cancers and related deaths occurred in China [2]. Unfortunately, in most cases, gastric cancer has already proceeded to advanced stage at the time of diagnosis, usually leading to poor prognosis, especially for those with unresectable gastric cancer [3]. However, barely any explicit definition or well‐recognized effective treatment for unresectable gastric cancer is available at present. Unresectable gastric cancer represents a huge unavoidable difficulty in the diagnosis and treatment. Encouragingly, conversion therapy could offer new opportunities for patients with unresectable gastric cancer. The aim of conversion therapy was to down‐stage primary tumor and/or metastatic lesions to achieve R0 resection. Several studies have demonstrated chemotherapy before surgery could achieve a relatively longer post‐operative survival for patients with primarily unresectable gastric cancer by obtaining partial or complete remission [4, 5, 6].

Local intensive therapy, represented by radiotherapy, shows significant efficacy in local tumor control and leads to a high R0 resection rate and good prognosis in locally advanced gastric cancer [7]. Interventional therapy, including transarterial infusion chemotherapy (TAIC), transarterial embolization (TAE), and transarterial chemoembolization (TACE), is another prospective mode of local intensive therapy for the treatment of tumors. As is well known, TACE with drug‐eluting bead (DEB) has become the standard treatment in advanced hepatocellular carcinoma as palliative treatment [8, 9]. In DEB‐TACE, microspheres carrying chemotherapeutic drugs are delivered, achieving sustained drug delivery, followed by embolization [10]. Many attempts have been made to clarify the feasibility and safety of interventional therapy in antitumor treatment for gastric cancer. TAE is often referred to as an alternative treatment in guidelines for patients with gastric cancer‐associated hemorrhage if it is failed to be controlled by endoscopy [11, 12, 13]. These patients are not suitable to receive systemic therapy or radiotherapy due to the lengthy preparation and delayed therapeutic effects. Although some of these patients may opt for endoscopic hemostasis, TAE provides a preferable choice for those with unstable hemodynamics and limited endoscopic visualization [14, 15]. Despite TAE has not been recommended as a standard in National Comprehensive Cancer Network (NCCN) and Chinese treatment guidelines yet, it is acknowledged that TAE had played a beneficial role in achieving hemostasis for gastric cancer [13, 16]. Besides, several previous studies have tried to use TAIC with systemic chemotherapy in late‐stage gastric cancer and demonstrated promising antitumor efficacy [17, 18, 19, 20, 21, 22]. The concepts of TAIC and TAE could be further integrated into TAICE. Currently, interventional therapies like TAICE or TAE have been recommended for patients with unresectable gastric cancer complicating with acute or subacute hemorrhage. Nevertheless, there is limited number of research in the clinical application of TAICE as peri‐operative therapy in the treatment of resectable or unresectable advanced gastric cancer, only with few case reports [23, 24, 25, 26].

From our observation of unresectable gastric cancer patients who received TAICE to control active tumor hemorrhage, we noticed that in some of these cases, the tumors exhibited significant shrinkage after the procedure. This unexpected finding led us to investigate the potential of utilizing TAICE as a newly emerging treatment strategy for unresectable gastric cancer. Thus, this retrospective study aimed to explore the value of TAICE in patients with unresectable locally advanced or metastatic cancer of stomach or gastroesophageal junction (GEJ).

## 2 Methods

### 2.1 Study Design and Population

This was a retrospective study at Zhongshan Hospital, Fudan University in China. Between January 2015 and December 2020, 27 patients with radiographically and pathologically confirmed treatment‐naive unresectable locally advanced or metastatic adenocarcinoma of stomach or GEJ who received TAICE for the treatment of tumor hemorrhage were included (Figure S1). All patients enrolled in this study achieved successful hemostasis for gastric cancer‐related bleeding through TAICE. The clinical success of hemorrhage control was defined as no rebleeding within 30 days after interventional therapy [14]. The exclusion criteria were (1) accompanied with other malignant tumors, (2) with cardiovascular events during or after interventional therapy, (3) benign ulcer, (4) Eastern Cooperative Oncology Group performance status ≥ 3, or (5) incomplete follow‐up data. These patients were preliminarily divided into two groups: locally advanced disease (cT4N + M0) and metastatic disease (cT4N + M1), based on the imaging and laparoscopic examinations. The unresectable factors were bulky lymph node metastasis, peritoneal metastasis, liver metastasis, retroperitoneal lymph node metastasis, adjacent organ or vascular involvement, portal vein tumor thrombus, and adrenal gland metastasis. The metastasis to distant organs, sites, or lymph node characterized M1 disease. Patients with unresectable factors encompassing the direct involvement of adjacent organs and blood vessels were categorized as those having locally advanced unresectable gastric cancer but still with a clinical stage of M0. The study was approved by the Ethics Committee of Zhongshan Hospital, Fudan University (IRB number: B2022‐332). The study protocol was in accordance with relevant guidelines and regulations or Declaration of Helsinki.

### 2.2 Treatment

TAICE was performed using the Seldinger method. The catheter was inserted through the femoral artery into the celiac artery. During TAICE, the catheter was first placed into the celiac artery for angiography to confirm the tumor feeding artery and tumor‐stained area. Then the catheter was further inserted into the tumor feeding artery such as left gastric artery or other arteries (e.g., gastroduodenal artery). The artery for infusion chemotherapy was selected by experienced radiologists. After the location of catheter was further determined by angiography, a certain dose of chemotherapeutic drugs was given by arterial infusion through catheter in the super‐selected tumor feeding artery. For patients with gastric cancer with more than one feeding arteries, infusion chemotherapy was performed within these arteries, respectively. The selection and dosage of chemotherapy regimen would be discussed and determined by the multidisciplinary team specializing in gastric cancer. In principle, concentration‐dependent drugs with reduced single dose were preferred. Then the gelatin sponge particles or microspheres were injected into the blood vessel for embolization. The microspheres were without drug loaded. The microspheres were Embosphere Microspheres from Merit Medical (South Jordan, UT, USA) with the diameter of 300–500 μm. The diameters of gelatin sponge were 350–760 μm. The catheter was withdrawn to the celiac trunk for a second‐time angiography. The endpoint of embolization could be considered as the tumor staining area completely or mostly disappeared under angiography. After interventional therapy, the possibility of surgery was evaluated by surgeons. Adjuvant therapy was administered for patients with gastrectomy, while later‐line therapy was given for those who could not undergo gastrectomy. TAICE regimen, adjuvant therapy, and later‐line therapy were prescribed at surgeons' discretion according to local guidelines.

### 2.3 Evaluations and Outcomes

Response to TAICE was evaluated using computed tomography (CT) according to the Response Evaluation Criteria In Solid Tumors, version 1.1 (RECIST 1.1) [27]. Pathological response was assessed using surgical specimens according to the Becker's tumor regression grade system [28]: grade 1a, no residual tumor cells; grade 1b, < 10% residual tumor cells; grade 2, 10%–50% residual tumor cells; and grade 3, > 50% residual tumor cells. During the follow‐up period, imaging examinations were performed every 6 months for patients who underwent gastrectomy and every 3 months for those without gastrectomy. Interventional treatment‐related adverse events (ITRAEs) were graded according to the National Cancer Institute Common Terminology Criteria for Adverse Events, version 5.0. Intraoperative and post‐operative complications were graded according to Clavien‐Dindo classification [29].

Outcomes included objective response rate (ORR, defined as the proportion of patients with complete response [CR] or partial response [PR]), disease control rate (DCR, defined as the proportion of patients with CR, PR, or stable disease [SD]), R0 resection rate (defined as the proportion of patients with R0 resection among patients with surgery), pathological complete response (pCR, no residual tumor cells) rate, major pathological response (MPR, < 10% residual tumor cells) rate, progression‐free survival (PFS, defined as time from the initiation of interventional therapy to disease recurrence, progression, or any‐cause death, whichever occurred first), overall survival (OS, defined as time from the initiation of interventional therapy to any‐cause death), and safety.

### 2.4 Statistical Analysis

Continuous variables were expressed as median (range), and categorical variables were expressed as frequency (percentage). PFS and OS were estimated using the Kaplan–Meier method, and their 95% confidence intervals (CIs) were calculated using the Brookmeyer–Crowley method. Comparisons of ORR, DCR, and incidence of ITRAEs among subgroups were performed using the chi‐square test, and comparisons of PFS and OS were performed using the log‐rank test. All the statistical analyses were performed using Stata software (version 17.0 [StataCorp LLC, Texas, USA]), R software, and GraphPad Prism (Version 9.5.0). *p* < 0.05 was considered statistically significant.

## 3 Results

### 3.1 Patient Characteristics

**FIGURE 1:**
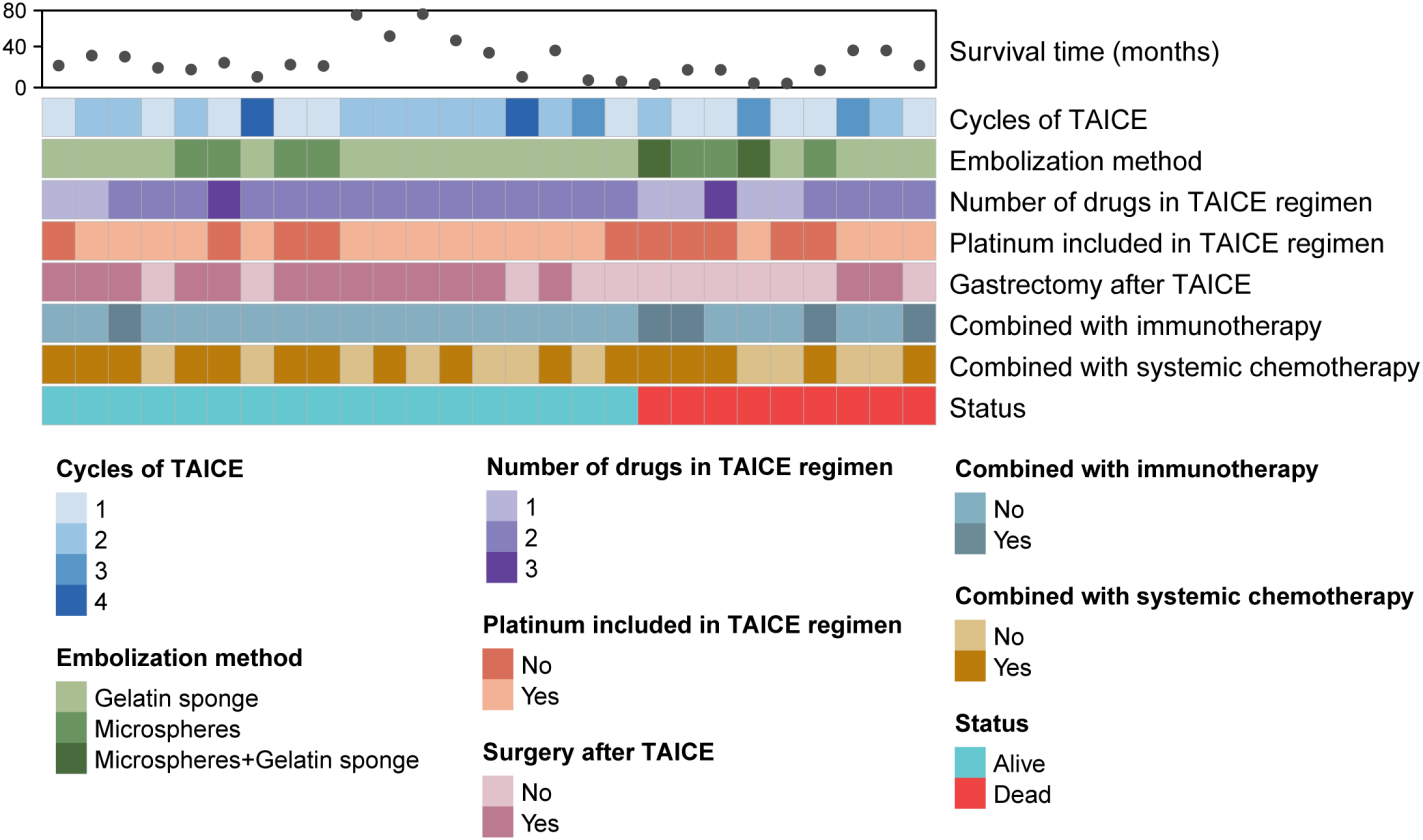
Heatmap of survival and clinicopathological information about TAICE therapy in 27 patients enrolled in this study.

A total of 27 patients were included in the final analysis (Figure S1). As presented in Table 1, the median age was 61 years (range, 34–83), and 19 (70.4%) patients were males. Nineteen (70.4%) patients had gastric adenocarcinoma, and eight (29.6%) had GEJ adenocarcinoma. Fourteen (51.9%) patients had unresectable locally advanced disease with a clinical stage of T4N + M0 (according to AJCC 8th edition), and 13 (48.1%) had metastatic lesions with a clinical stage of T4N + M1. The unresectable factors were as follows: bulky lymph node metastasis (15 [41.7%]) [30], peritoneal metastasis (4 [11.1%]), liver metastasis (5 [13.9%]), retroperitoneal lymph node metastasis (4 [11.1%]), adjacent organ or vascular involvement (6 [16.6%]), portal vein tumor thrombus (1 [2.8%]), and adrenal gland metastasis (1 [2.8%]). Less than half (37.0%) of the patients had more than two unresectable factors. The detailed information about TAICE therapy for 27 patients in this study is exhibited in Figure 1. All of the patients enrolled in this study were diagnosed with gastric cancer and received TAICE for the control of tumor hemorrhage. The median number of TAICE cycles was 2 (range, 1–4). More than half (59.3%) of the patients received TAICE combined with systemic chemotherapy, but only few of them (18.5%) received TAICE combined with immunotherapy (Tables S1 and S2). Platinum was the most commonly used drug in TAICE regimen (63.0% [17/27]) and was likely related to a satisfactory long‐term oncological effect like OS. Following TAICE, 15 (55.6%) of 27 patients underwent gastrectomy and D2 lymph node dissection.

**TABLE 1:** Patient characteristics.

Characteristic	Patients (*n* = 27)
Age, years, median (range)	61 (34–83)
Sex, *n* (%)	
Male	19 (70.4)
Female	8 (29.6)
Primary tumor site, *n* (%)	
Stomach	19 (70.4)
Gastroesophageal junction	8 (29.6)
Tumor size, cm, median (range)	6.8 (3.5, 17.4)
Clinical stage, *n* (%)	
T4N + M0	14 (51.9)
T4N + M1	13 (48.1)
Lauren's type, *n* (%)	
Intestinal	11 (40.7)
Mixed	10 (37.0)
Diffuse	6 (22.3)
Number of unresectable factors, *n* (%)	
1	17 (63.0)
≥ 2	10 (37.0)
Unresectable factor, *n* (%)	
Bulky lymph node metastasis	15 (41.7)
Peritoneal metastasis	4 (11.1)
Liver metastasis	5 (13.9)
Retroperitoneal lymph node metastasis	4 (11.1)
Adjacent organ or vascular involvement	6 (16.6)
Portal vein tumor thrombus	1 (2.8)
Adrenal gland metastasis	1 (2.8)

### 3.2 Response to TAICE

**FIGURE 2:**
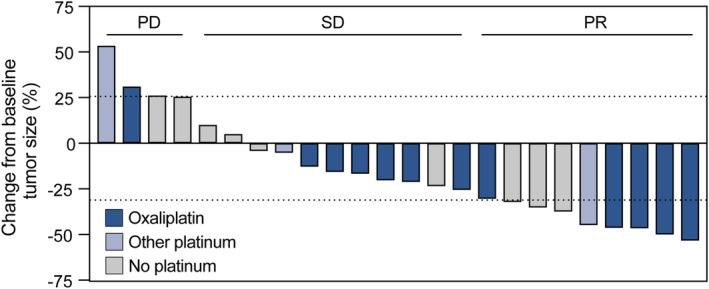
Waterfall plot of best overall response in 24 patients with evaluable response. PD, progressive disease; SD, stable disease; PR, partial remission.

**FIGURE 3:**
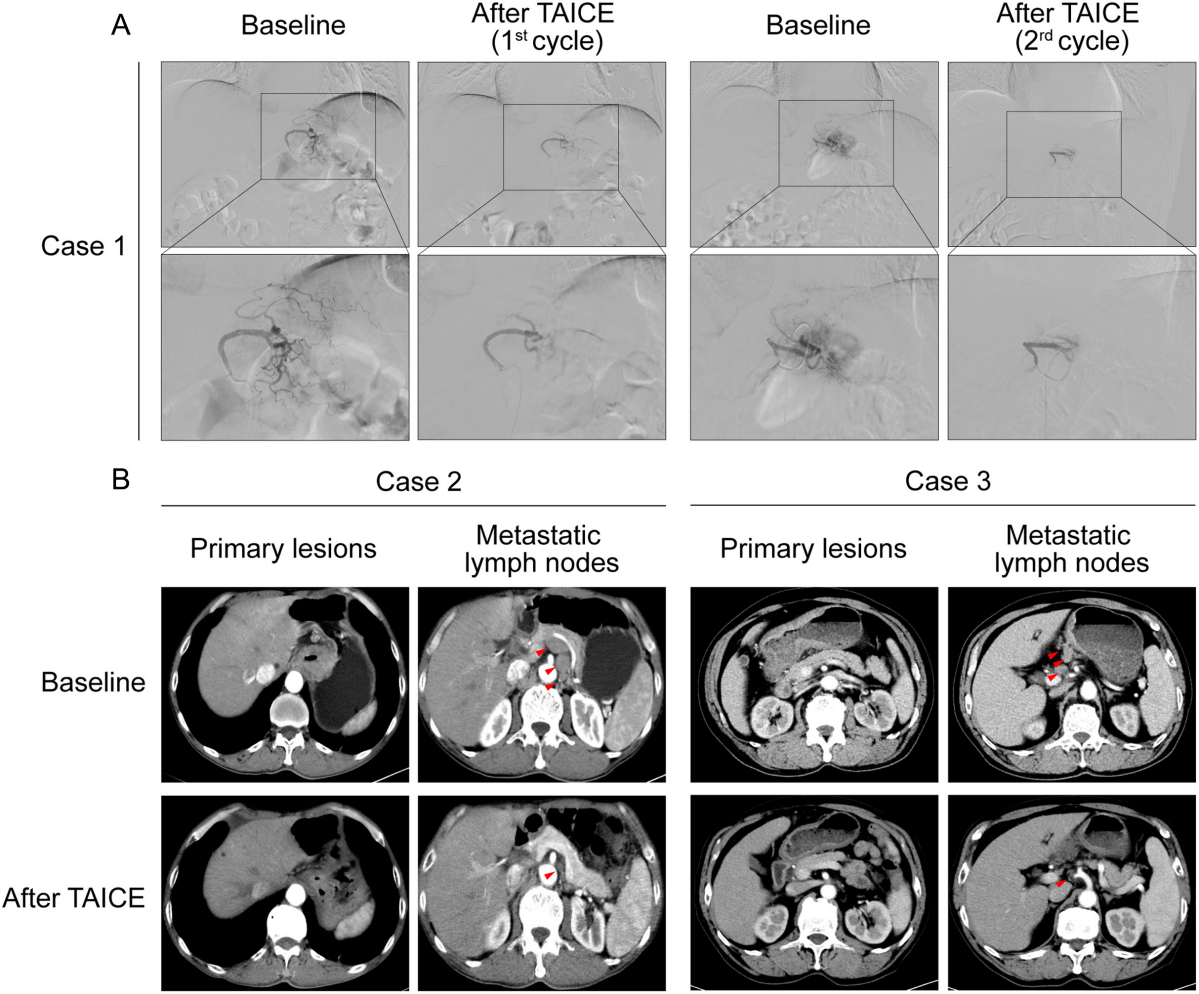
(A) Representative digital subtraction angiography (DSA) images of tumor feeding artery of gastric cancer from one patient at baseline and after TAICE in two cycles of TAICE. (B) Representative computed tomography (CT) images of primary lesions and lymph node metastases of gastric cancer from two patients at baseline and after TAICE. Red arrows indicated lymph node metastases.

Of 27 patients, the ORR was 33.3%, and the DCR was 74.0%. Subgroup analyses showed that patients who received TAICE with platinum in regimen had numerically higher ORR and DCR than those without platinum (35.3% vs. 30.0%, 76.4% vs. 70.0%, respectively, Table 2). Waterfall plot of best change in tumor size from baseline is shown in Figure 2. Patients who received TAICE with platinum especially oxaliplatin were proned to obtain a significant shrinkage of tumor size. During TAICE, digital subtraction angiography (DSA) was utilized for the selection of the targeted tumor feeding artery such as left gastric artery. It was also confirmed by DSA that the successful procedure of infusion chemotherapy and embolization (Figure 3A). Intriguingly, neovascularization of tumor feeding artery was observed at the baseline evaluation of the second cycle of TAICE by DSA. Representative changes in primary lesions and lymph node metastases by CT from two patients are shown in Figure 3B. A significant remission of primary lesions of gastric cancer after TAICE could be intuitively observed. Additionally, we found that in some patients with peri‐gastric lymph node metastases and retroperitoneal lymph node metastases at the diagnosis, TAICE for primary lesions of gastric cancer could also exert an effect on lymph nodal metastases to a degree.

**TABLE 2:** Response to TAICE.

Variable	Total (*n* = 27)	No platinum (*n* = 10)	Platinum (*n* = 17)
Best response, *n* (%)			
Complete response	0	0	0
Partial response	9 (33.3)	3 (30.0)	6 (35.3)
Stable disease	11 (40.7)	4 (40.0)	7 (41.1)
Progressive disease	4 (14.9)	2 (20.0)	2 (11.8)
Not evaluated	3 (11.1)	1 (10.0)	2 (11.8)
ORR, %	33.3	30.0	35.3
DCR, %	74.0	70.0	76.4
Gastrectomy after TAICE, *n*/%	15/55.6	4/40.0	11/64.7
OS, median (95%CI)	36.1 (21.0–NR)	16.6 (2.4–NR)	Undefined (36.0–NR)
PFS, median (95%CI)	19.8 (12.1–40.0)	14.7 (0.9–NR)	33.2 (12.1–NR)

Abbreviations: CI, confidence interval; DCR, disease control rate; NR, not reached; ORR, objective response rate; OS, overall survival; PFS, progression‐free survival.

### 3.3 Surgical Results

**FIGURE 4:**
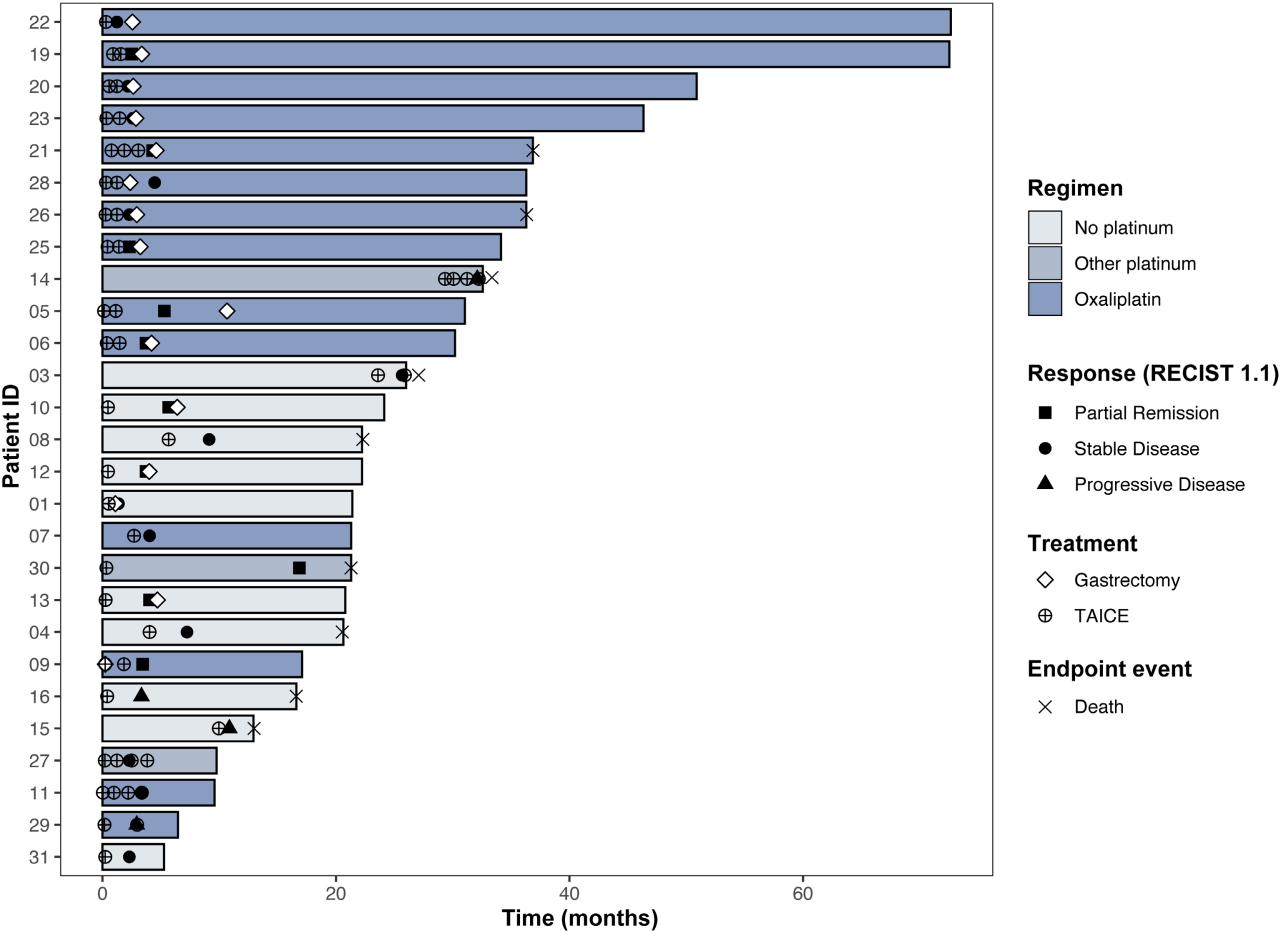
Swimming plot demonstrating events during treatment and follow‐up in 27 patients.

Eighteen patients underwent surgery following TAICE, and three of them only received surgical exploration without gastrectomy due to intra‐abdominal metastasis. Of 15 patients with gastrectomy (Figure 4), five (33.3%) had distant metastasis before TAICE. Surgical exploration found complete remission (CR) at peritoneal metastasis in one of these five patients, and imaging examinations found CR at distant metastasis in other four patients. (liver metastases, bulky lymph node metastasis, and portal vein tumor thrombus) in the other four patients. The R0 resection rate was 83.3% (15/18). Seven (46.7%) patients had tumor down‐staging from N+ to N0. Four (26.7%) patients achieved MPR, but no patients achieved pCR (Table 3).

**TABLE 3:** Characteristics of patients who underwent surgery.

Characteristic	Patients (*n* = 15)
Age, years, median (range)	61 (40–76)
Sex, *n* (%)	
Male	10 (66.7)
Female	5 (33.3)
Clinical stage, *n* (%)	
T4N + M0	10 (66.7)
T4N + M1	5 (33.3)
Number of interventional therapy cycles, median (range)	2 (1–3)
Surgery, *n* (%)	
Proximal	1 (6.7)
Distal	8 (53.3)
Total	6 (40.0)
Number of dissected lymph nodes, median (range)	40 (27–55)
ypT stage, *n* (%)	
T1	4 (26.7)
T2	1 (6.7)
T3	5 (33.3)
T4	5 (33.3)
ypN stage, *n* (%)	
N0	7 (46.7)
N+	8 (53.3)
Becker's tumor regression grade, *n* (%)	
Grade 1a	0
Grade 1b	4 (26.7)
Grade 2	3 (20.0)
Grade 3	7 (46.7)
Not evaluable	1 (6.6)
Pathological complete remission, *n* (%)	0
Major pathological remission, *n* (%)	4 (26.7)

### 3.4 Survival

**FIGURE 5:**
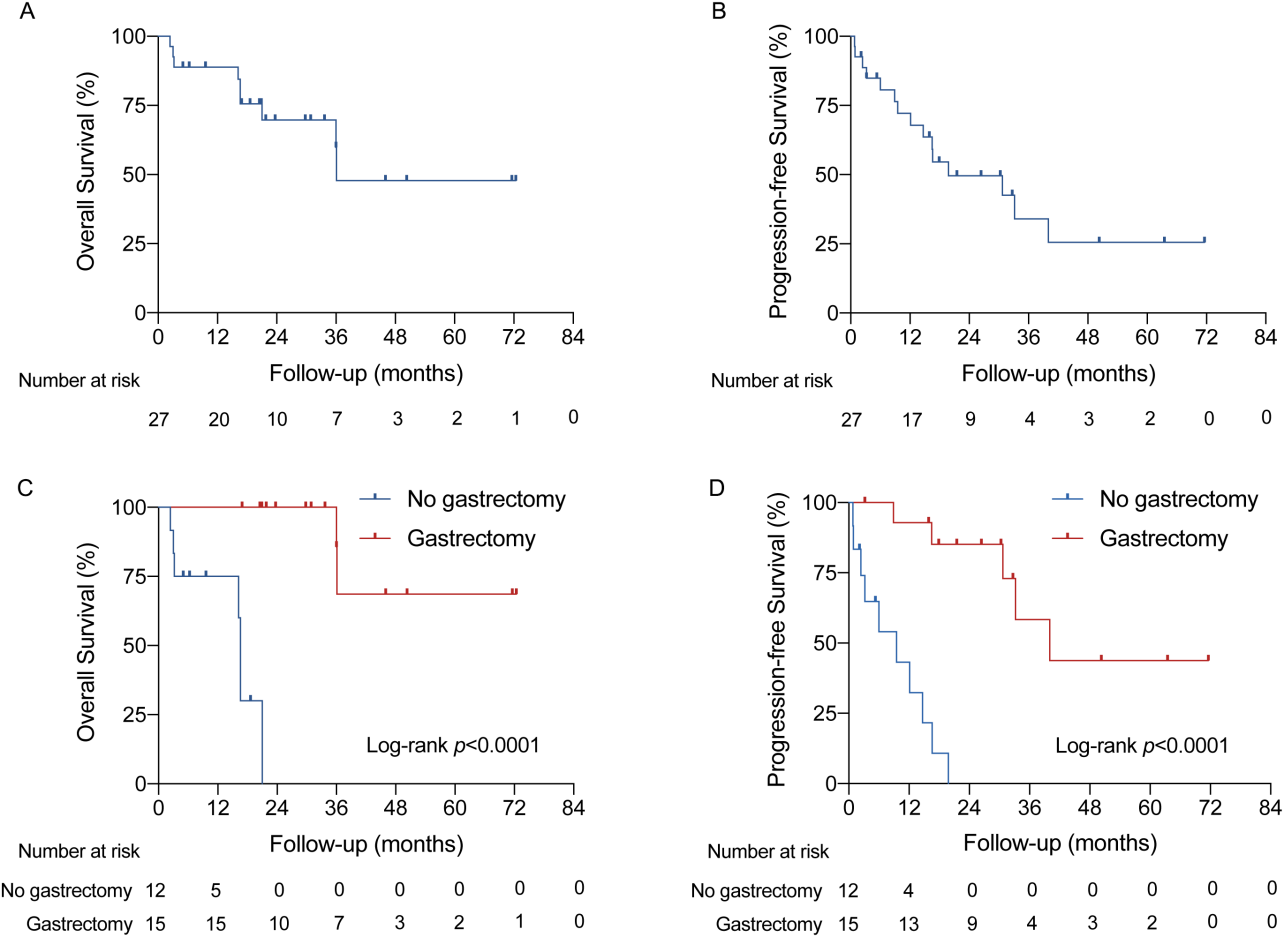
Kaplan–Meier curves for survival. (A) Overall survival in 27 patients; (B) Progression‐free survival in 27 patients; (C) Overall survival in patients with or without surgery; (D) Progression‐free survival in patients with or without surgery.

With a median follow‐up of 29.8 months (range, 2.4–72.4), the median PFS was 19.8 months (95%CI, 12.1–40.0), and the median OS was 36.1 months (95%CI, 21.0–NR [not reached]) among 27 patients (Table 2 and Figure 5A,B). Subgroup analyses showed that patients with gastrectomy had significantly longer PFS (40.0 vs. 9.5 months, *p* < 0.0001) and OS (NR vs. 16.6 months, *p* < 0.0001) than those without gastrectomy (Figure 5C,D). TAICE regimen with platinum resulted in numerically longer PFS (33.2 vs. 14.7 months, *p* = 0.096) and significantly longer OS (NR vs. 16.6 months, *p* = 0.019) than regimen without platinum (Table 2 and Figure S2).

### 3.5 Safety

ITRAEs were observed in all the 27 patients, which were all grade 1 or 2 (Table 4). This suggested a favorable safety profile with TAICE. The most common ITRAEs were fatigue (27 [100.0%]), nausea (17 [63.0%]), vomiting (15 [55.6%]), and fever (6 [22.2%]). No sensory neuropathy occurred during TAICE, and no severe surgical complications occurred. Six (33.3%) of 18 patients with gelatin sponge embolization had fever (not febrile neutropenia), while no fever events occurred in seven patients with microspheres embolization. Similarly, only patients with gelatin sponge embolization had abdominal pain (2 [11.1%]) requiring symptomatic treatment or increased AST/ALT (6 [33.3%]), while no such ITRAEs were observed in patients with microspheres embolization, which indicated a better safety of TAICE with microspheres embolization.

**TABLE 4:** Interventional treatment‐related adverse events (*n* = 27).

Event	Any grade, *n* (%)	Grade 1, *n* (%)	Grade 2, *n* (%)
Fatigue	27 (100.0)	24 (88.9)	3 (11.1)
Nausea	17 (63.0)	3 (11.1)	14 (51.9)
Vomiting	15 (55.6)	12 (44.4)	3 (11.1)
Fever	6 (22.2)	6 (22.2)	0
Abdominal pain	2 (7.4)	2 (7.4)	0
AST and/or ALT increased	6 (22.2)	6 (22.2)	0

*Note:* No grade 3–5 adverse events occurred.

Abbreviations: ALT, alanine aminotransaminase; AST, aspartate aminotransferase.

## 4 Discussion

This study retrospectively explored the feasibility and safety of TAICE in patients with unresectable locally advanced or metastatic cancer of stomach or GEJ. Basically, the results suggested that TAICE was a safe approach and could provide an opportunity of surgery for these patients, leading to good survival outcomes. Originally, all patients included in this study received TAICE as treatment for tumor‐related hemorrhage. Surprisingly, remarkable tumor regression was noticed during the treatment, with an ORR of 33.3% and DCR of 74.0%. Moreover, approximately half (55.6%) of patients with unresectable lesions were successfully converted to accept radical surgery and achieved a relatively high R0 resection rate of 83.3% (15/18). The short‐term effects such as tumor remission and the R0 resection rate after TAICE indicated the potential value of TAICE as a feasible therapy for down‐staging unresectable gastric cancer. Meanwhile, in our study, patients who received the combination of TAICE and subsequent radical surgery indicated survival benefit. The median PFS was 19.8 months (95%CI, 12.1–40.0), and the median OS was 36.1 months (95%CI, 21.0–NR) among 27 patients. Compared with patients without gastrectomy, patients who received gastrectomy after TAICE had a significantly longer PFS (40 months vs. 9.5 months, *p* < 0.0001) and OS (NR vs. 16.6 months, *p* < 0.0001). Yet, there is no high‐level evidence or guidelines existing to support the pre‐operative use of TAICE for the treatment of gastric cancer. More efforts need to be paid to make a solid conclusion on the significance of clinical application of TAICE in unresectable gastric cancer pre‐operatively.

Our study stressed that interventional therapy could be a potentially effective approach for the treatment of unresectable gastric cancer. While limited by the nature of single‐arm study, the direct comparison of effect between systemic chemotherapy and interventional therapy could not be conducted in this study. However, by reviewing the data of published literature, our results suggested that TAICE might lead to good survival outcomes [13]. In a previous phase 2 trial, patients with unresectable advanced gastric cancer received a median of five cycles of intravenous chemotherapy [31]. The surgery rate was 34.9%, the median PFS was 11.7 months, and the median OS was 24.1 months. Patients in our study received a median of two cycles of TAICE, but the surgery rate was 55.6%, with longer median PFS (19.8 months) and OS (36.1 months). We speculated that interventional therapy had stronger local effect on locally advanced tumor than systemic chemotherapy.

Although TAICE or other interventional therapies like TAIC and TAE are generally applied in palliative treatment of late‐stage gastric cancer and have been written in guidelines [32] so far, no standardized regimen has been brought up and widely approved. According to previous reports, two cycles of TAIC combined with intravenous chemotherapy showed a higher surgery rate (68.1%–74.3%), but lower median OS (18–23 months), compared with our results [17, 19, 20]. This gave a hint that the embolization might be essential for the long‐term effect of TAICE. Meanwhile, our results also revealed a better safety of TAICE with microspheres embolization with a lower incidence of ITRAEs. Another matter of concern is the option of drugs in TAICE regimen. Platinum shows antitumor activity in a concentration‐dependent manner. Intravenous administration limits the total dose of drug given its toxicity. Infusion through selected artery can reduce the first‐pass effect and elevate the local concentration of drug without increasing the total dose and toxicity [33]. In our study, we found that patients treated with platinum could result in better OS than those without platinum, and the most commonly used platinum was oxaliplatin. The role of platinum‐based regimen in TAICE and the optimal option of platinum need to be verified in future studies.

Despite encouraging findings in our study, we also acknowledged some limitations. First, the nature of retrospective study inevitably leads to confounding bias and selection bias. Confounding factors such as TNM stage or pathological type which are closely related to the short‐term and long‐term oncological effectiveness of the treatment were hard to control by stratification analysis. Our study applied non‐random sampling with no matching control group, which inevitably leaded to selection bias. Second, considering the different clinical settings across studies and small population of our study, large‐scale prospective studies need to be designed in further research to verify the conclusion of this study. Third, the contribution of systemic chemotherapy in the entire effect of treatment was unable to be measured in this study. The interaction between systemic chemotherapy and TAICE could not be revealed as well. In addition, multivariate analysis could not be performed also due to a relatively small sample size.

In conclusion, TAICE could be a potential new strategy to provide opportunity of surgery for patients with unresectable advanced gastric or GEJ cancer, leading to improved survival. A prospective study of pre‐operative TAICE in locally advanced adenocarcinoma of stomach or GEJ is ongoing in our center (NCT05396326).

## Author Contributions

**Lingqiang Min:** data curation (equal), formal analysis (equal), writing – original draft (equal), writing – review and editing (equal). **Zheng Liu:** data curation (equal), visualization (equal), writing – original draft (equal), writing – review and editing (equal). **Bo Zhou:** data curation (equal), formal analysis (equal). **Peng Zhou:** data curation (equal), formal analysis (equal). **Rongkui Luo:** data curation (equal), formal analysis (equal). **Yuqin Ding:** data curation (equal), formal analysis (equal). **Yuehong Cui:** data curation (equal), formal analysis (equal). **Zhongyi Shi:** data curation (equal), formal analysis (equal). **Yuan Gu:** data curation (equal), formal analysis (equal). **Yihong Sun:** data curation (equal), formal analysis (equal), resources (equal). **Zhaoqing Tang:** conceptualization (equal), resources (equal), supervision (equal), writing – original draft (equal), writing – review and editing (equal). **Xuefei Wang:** conceptualization (equal), resources (equal), supervision (equal), writing – review and editing (equal).

## Ethics Statement

This study was approved by the Ethics Committee of Zhongshan Hospital, Fudan University (IRB number: B2022‐332).

## Consent

The need for written informed consent was waived due to the retrospective nature of the study by the Ethics Committee of Zhongshan Hospital, Fudan University.

## Conflicts of Interest

The authors declare no conflicts of interest.

## Supporting information


**FIGURE S1.** Patient enrollment flowchart. ECOG, Eastern Cooperative Oncology Group.

**FIGURE S2.** Kaplan–Meier curves for survival. (A) Overall survival in 27 patients with or without platinum in TAICE regimen; (B) Progression‐free survival in 27 patients with or without platinum in TAICE regimen.

**TABLE S1.** The peri‐operative therapies (except TAICE) of enrolled patients.
**TABLE S2.** Systemic chemotherapy regimen of enrolled patients.

## Data Availability

All data generated or analyzed during this study are included in this published article.
