# Supplementary material for: Transarterial Infusion Chemotherapy and Embolization for Patients With Unresectable Advanced Cancer of Stomach or Gastroesophageal Junction: A Retrospective Study
Source: Cancer Med. 2024 Nov 5;13(21):e70396. doi: 10.1002/cam4.70396 (PMC11536461; doi:10.1002/cam4.70396)
Supplement: Supplementary file 1 — FIGURE S1. Patient enrollment flowchart. ECOG, Eastern Cooperative Oncology Group. [file CAM4-13-e70396-s003.pdf]

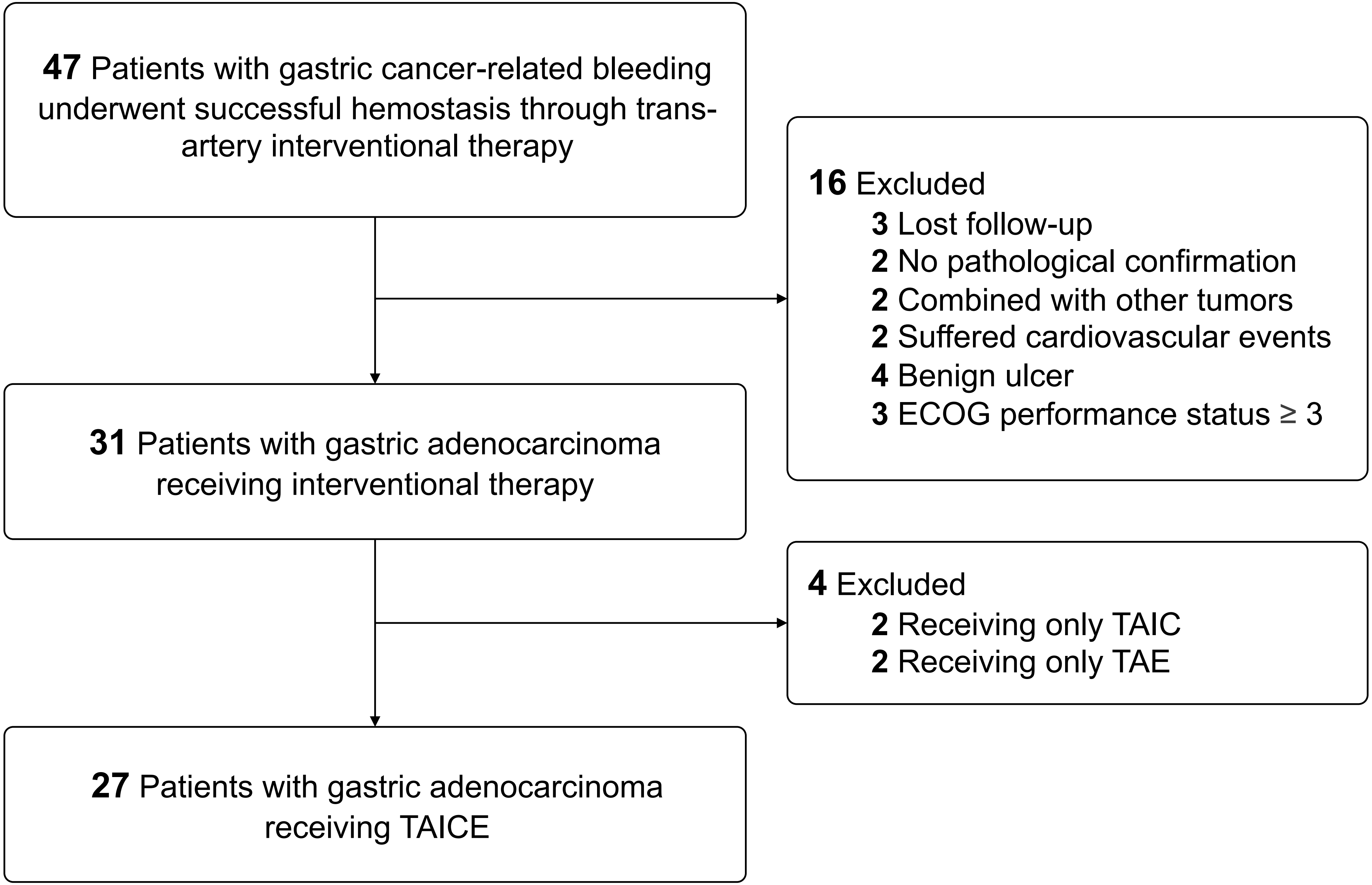
47 Patients with gastric cancer-related bleeding underwent successful hemostasis through trans-artery interventional therapy
16 Excluded
3 Lost follow-up
2 No pathological confirmation
2 Combined with other tumors
2 Suffered cardiovascular events
4 Benign ulcer
3 ECOG performance status ≥ 3
31 Patients with gastric adenocarcinoma receiving interventional therapy
4 Excluded
2 Receiving only TAIC
2 Receiving only TAE
27 Patients with gastric adenocarcinoma receiving TAICE
