# Supplementary figures and images for: Transarterial Infusion Chemotherapy and Embolization for Patients With Unresectable Advanced Cancer of Stomach or Gastroesophageal Junction: A Retrospective Study
Source: Cancer Med. 2024 Nov 5;13(21):e70396. doi: 10.1002/cam4.70396 (PMC11536461; doi:10.1002/cam4.70396)

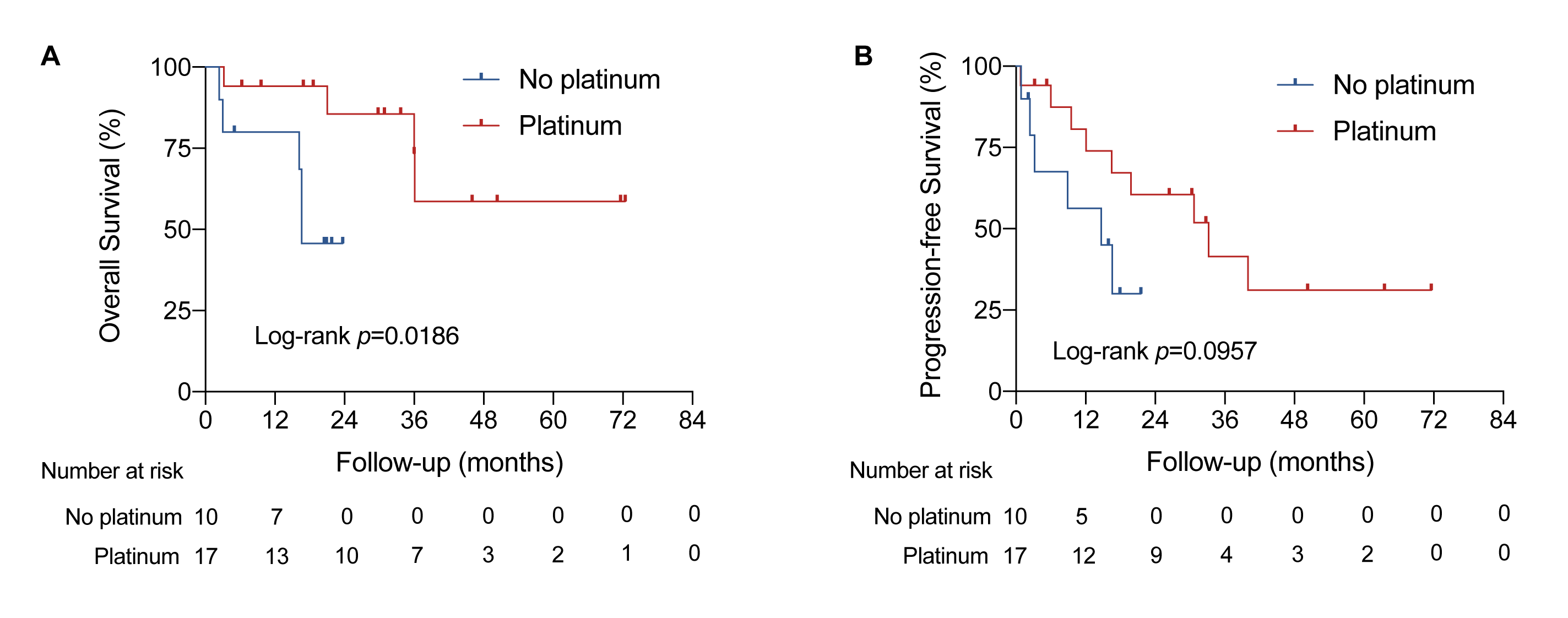

Supplement: Supplementary file 2 — FIGURE S2. Kaplan–Meier curves for survival. (A) Overall survival in 27 patients with or without platinum in TAICE regimen; (B) Progression‐free survival in 27 patients with or without platinum in TAICE regimen. [file CAM4-13-e70396-s002.tif]
