# Supplementary material for: Transarterial Infusion Chemotherapy and Embolization for Patients With Unresectable Advanced Cancer of Stomach or Gastroesophageal Junction: A Retrospective Study
Source: Cancer Med. 2024 Nov 5;13(21):e70396. doi: 10.1002/cam4.70396 (PMC11536461; doi:10.1002/cam4.70396)
Supplement: Supplementary file 3 — TABLE S1. The peri‐operative therapies (except TAICE) of enrolled patients. TABLE S2. Systemic chemotherapy regimen of enrolled patients. [file CAM4-13-e70396-s001.docx]

**Supplementary Table 1. The peri-operative therapies (except TAICE) of enrolled patients**

| Therapy | Status | Patients (n=27) | Proportion |
| --- | --- | --- | --- |
| Peri-operative therapy | Yes | 16 | 59.26% |
|  | No | 11 | 40.74% |
| Systemic chemotherapy | Yes | 16 | 59.26% |
|  | No | 11 | 40.74% |
| Targeted therapy | Anti HER-2 | 3 | 11.11% |
|  | Anti VEGF | 1 | 3.70% |
|  | No | 23 | 85.19% |
| Immunotherapy | Anti PD-1 | 5 | 18.52% |
|  | No | 22 | 81.48% |

Abbreviations: PD-1, programmed cell death protein-1; VEGF, vascular endothelial growth factor

**Supplementary Table 2. Systemic chemotherapy regimen of enrolled patients**

|  | Regimen | Count |
| --- | --- | --- |
| Multiple drug regimen | FOLFOX | 1 |
|  | RALOX | 2 |
|  | XELOX | 2 |
|  | DOS | 3 |
|  | SOX | 3 |
|  | TS | 2 |
|  | IP | 1 |
|  | AS | 1 |
| Single drug regimen | Abraxane | 2 |
|  | Irinotecan | 1 |
|  | S-1 | 4 |
|  | Docetaxel | 2 |
|  | Capecitabine | 1 |

**Detailed regimen**:

S-1: Tegafur Gimeracil Oteracil Potassium

FOLFOX: Oxaliplatin, Calcium folinate, 5-fluorouracil

RALOX: Oxaliplatin, Raltitrexed

XELOX: Oxaliplatin, Capecitabine

DOS: Docetaxel, Oxaliplatin, S-1

SOX: Oxaliplatin, S-1

TS: Taxol, S-1

IP: Irinotecan, Cisplatin

AS: Abraxane, S-1
